# Reducing Anxiety in Stutterers through the Association between “Purpose in Life/Ikigai” and Emotions

**DOI:** 10.5539/gjhs.v4n5p120

**Published:** 2012-08-09

**Authors:** Riichiro Ishida

**Keywords:** purpose in life/ikigai, social desirability, stuttering, anxiety, serotonin, dopamine

## Abstract

The prefrontal lobe is more evolved in humans than in other mammals. The functioning of human prefrontal lobes promotes an innate need to establish a meaningful life, often referred to as “Purpose in life (PIL)/ikigai.” PIL/ikigai and the prefrontal lobe psychologically and physiologically, respectively, shape ambition, regulate the development of emotions and integrate psychological events. PIL/ikigai contributes to both a reduction in the anxiety caused by a need for approval from others and the stimulation of pleasure and comfort, which may be related to the well-balanced secretion of neurotransmitters such as serotonin, dopamine, and β-endorphin. Thus, if a stutterer aware that articulate speech is advantageous in communication feels a need for social desirability (SD) based on a need for approval from others, they may develop stress and anxiety caused by their perceived failure when engaging in conversations. This feeling of failure may be related to an imbalanced secretion of serotonin and dopamine. Therefore, previous work has suggested that PIL/ikigai may reduce anxiety in stutterers who seek SD by reducing the symptoms associated with stuttering.

## 1. Introduction

Various types of stress may cause anxiety, which in turn may lead to the development of psychiatric and somatic diseases including depression and cardiovascular disease (Hosaka, 2012; Ishida & Okada, 2011; H Jozuka, E Jozuka, Suzuki, Takeuchi, & Takatsu, 2003; Shioiri, Someya, Murashita, & Takahashi, 1996; Smith, Nolen-Hoeksema, Fredrickson, & Loftus, 2003). Stuttering is a speech disorder that may be exacerbated by the stress of human communication (Disord, 2005; Friedlander, Noffsinger, Mendez, & Yagiela, 2004; Costa & Krol, 2000; Freeman & Silver, 1989). Most stutterers are said to have anxiety before and during conversations because of their perceived negative evaluation from others (Disord, 2005; Friedlander et al., 2004; Costa & Krol, 2000; Freeman & Silver, 1989; Murray, 1985). Their anxiety is exacerbated by repeated failure, i.e., stuttering, which may then lead to depression and withdrawal (Freeman & Silver, 1989; Murray, 1985). Social desirability (SD), a feeling that promotes people to adapt to a social expectation perceived as valuable, is based on approval motivation (Ishida, 2008a). SD develops through negative experiences, such as excessive expectations placed on children by parents and/or teachers from infancy through adolescence (Ishida, 2008a), and thus stutterers may develop an even more excessive form of SD due to their additional difficulties in communicating articulately. However, some stutterers experience diminished anxiety and effectively contribute to society regardless of their condition (Murray, 1985). Therefore, the anxiety caused by stuttering is more important than the actual symptoms of stuttering. Accordingly, various psychological and physiological techniques for reducing stuttering have been developed (Freeman & Silver, 1989). For example, studies have examined the effects of respiration speech training and oral muscle training (Freeman & Silver, 1989). However, the alleviation of symptoms from these techniques is often only temporary. Thus, a fundamental way to reduce anxiety in stutterers should be developed.

Recently, studies have demonstrated that development of purpose in life (PIL)/ikigai as a social attitude is achieved through positive experiences, including enjoying the beauty of nature and the outdoors and other natural and effective ways to cope with stress (Ishida, 202a). SD, by contrast, causes anxiety during stressful situations that involve evaluation from others (Ishida, 2008a). Cognitive researchers investigating prefrontal lobe functions of the brain, as well as the chemical traits of neurotransmitters controlling emotions, support the significance of PIL/ikigai and the problem of SD (Ishida, 2012a; Solomon, Berg, & Martin, 2011; Smith et al., 2003; Craik & Grady, 2002). Therefore, we hypothesize that PIL/ikigai may reduce anxiety in stutterers with the need for SD and thus lead to a reduction in stuttering symptoms. The present study will discuss the significance of PIL/ikigai based on previous psychological and physiological evidence including motivation, brain function, and neurotransmitters (Craik & Grady, 2002; Frankl, 1972; Freeman & Silver, 1989; Ishida, 2012a: Ishida, 2012b; Ishida & Okada, 2011; Kamiya, 2004; Smith et al., 2003; Solomon et al., 2011).

## 2. Mechanisms of Stuttering and Purpose in Life (PIL)/Ikigai

It is important to understand the link between the state of mind and the body. Therefore, the mechanism involved in stuttering and its relation to PIL/ikigai and SD will be discussed within the context of brain function and the chemical traits of the neurotransmitters that control emotion.

### 2.1 Prefrontal Lobe and Neurotransmitters

The prefrontal lobe, which connects extensively with other areas of the brain, is more evolved in humans than in other mammals (Ishida, 2012a), and contributes to the development and regulation of emotion, motivation, ambition, response to stress, evaluation of information, integration of psychological events, judgments and decisions, planning, and organization of response (Solomon et al., 2011; Smith et al., 2003; Craik & Grady, 2002). In turn, all of these functions depend on neurotransmitters (Solomon et al., 2011; Smith et al., 2003). In particular, dopamine has been demonstrated to be related to motivation, while noradrenaline has been linked to anxiety, and β-endorphin has been related to a decrease in pain and an increase in comfort. Moreover, serotonin has been shown to control the well-balanced secretions of these neurotransmitters and those related to overall comfort (Solomon et al., 2011; Smith et al., 2003). Previous research has suggested that the processes involved in experiences that lead to learning and the development of memories differ among individuals; and these processes may change neuronal networks through the repeated secretion of neurotransmitters (Solomon et al., 2011). Therefore, evidence for an association among the mind, brain function, and neurotransmitters has been observed.

### 2.2 Stuttering

Stuttering consists of core respiratory/laryngeal/articulatory spasms and incoordination associated with learned (secondary) behaviors (Freeman & Silver, 1989). Anxiety in stutterers, caused by a fear of failure when speaking, results from a need for approval from others, as well as the avoidance of punishment (Freeman & Silver, 1989; Murray, 1985). People feel stress, which in turn may cause anxiety, confusion, and tension, when they cannot predict future outcomes (Smith et al., 2003). Stutterers with excessive SD develop traits such as anxiety, confusion, and tension through experienced failure in situations where they have to engage in conversations (Craik & Grady, 2002). Anxiety and other symptoms of stuttering are exacerbated by repeated failure in situations where they have to speak with others (Freeman & Silver, 1989). Thus, both the need for approval and the avoidance of punishment from others are also elevated (Craik & Grady, 2002; Murray, 1985). This overall process also strengthens neuronal networks through an imbalanced secretion of neurotransmitters, including serotonin and dopamine (Disord, 2005; Friedlander et al., 2004; Costa & Krol, 2000). Consequently, the strengthened and continual feelings of anxiety influence both internal secretion (Selye, 1936; 1973) and the autonomic function controlling homeostatic functioning (Cannon, 1939) resulting in development of additional diseases and disorders in stutterers. This suggests that the anxiety associated with future occurrences of stuttering, which is related to learned behaviors, is an important problem for stutterers. Therefore, development of PIL/ikigai may be an effective strategy for reducing anxiety in stutterers and helping them cope with stress.

### 2.3 Purpose in Life (PIL)/Ikigai

The phrase PIL originates from 19^th^ century European existential philosophy (Kierkegaard, 1844), while the term ikigai can be traced back to 14^th^ century Japan (Goto & Kamada, 1960). Both terms commonly propose that “Everything changes. Life is a one-time only opportunity. Every person has a natural and intrinsic need to achieve meaning in their life” (Kamiya, 2004; Frankl, 1972). Taken together, these phrases indicate that PIL/ikigai is psychologically related to a view of ambition in life. The prefrontal lobe is physiologically related to functions such as ambition, regulation of emotion, and integration of psychological events (Solomon et al., 2011; Smith et al., 2003; Craik & Grady, 2002). Therefore, PIL/ikigai may be linked to prefrontal lobe functioning (Ishida, 2012a). PIL/ikigai integrates psychologically stressful events from the past, present, and potential future and enables individuals to associate these experiences with less anxiety and confusion, even during stressful situations (Ishida, 2012b; Ishida & Okada, 2011). For example, persons who have achieved PIL/ikigai feel less anxious during conversations with others (Ishida, 2008b). The integration mechanism of PIL/ikigai is similar to the “law of Prägnanz,” a Gestalt psychological concept that proposes a mechanism for integrating visual figures (Kurato, 2011). The successful integration of an individual’s PIL/ikigai leads to a well-balanced secretion of emotion-related neurotransmitters such as dopamine, noradrenaline, β-endorphin and serotonin (Solomon et al., 2011; Smith et al., 2003). Previous research has demonstrated that PIL/ikigai is negatively correlated with SD, the need for approval from others and anxiety (Ishida, 2008a; Ishida, 2012a: Ishida & Okada, 2011). Case studies have shown that the development of social attitudes, i.e., view of life, is an important factor in reducing symptoms of stuttering (Murray, 1985). People who have developed PIL/ikigai are able to delay gratification, appreciate points of view of others, hold religious beliefs, accept personal limitations and appreciate personal blessings (Ishida, 2012a). Creating new experiences leads to the repeated secretion of neurotransmitters and causes changes in neuronal networks (Solomon et al., 2011). These findings suggest that development of PIL/ikigai in stutterers may fundamentally reduce the anxiety caused by a prediction of failure when speaking with others, which stems from a need for approval. As previous studies have found (e.g., Freeman & Silver, 1989), this process may lead to a decrease in symptoms of stuttering, and thus stuttering associated with learned secondary behaviors may be relatively easy to extinguish.

### 2.4 Developing PIL/Ikigai (A Brief Note)

It is useful for both stutterers and non-stutterers to know how to develop PIL/ikigai. PIL/ikigai can be developed through positive experiences from infancy throughout adolescence, and may be able to provide a person with comfort, pleasure, less anxiety, and a well-balanced secretion of neurotransmitters (e.g., Ishida, 2012a). These experiences include the enjoyment of nature and the outdoors, sympathetic acceptance from others, including parents and teachers, and the positive overall impressions made by people and events (e.g., Ishida, 2012a). Thus, repeated positive experiences may serve as intrinsic rewards which psychologically strengthen PIL/ikigai (Ishida, 2012a), and thus physiologically strengthen neuronal networks through the repeated secretion of neurotransmitters (Solomon et al., 2011). The excessive need for approval from others, by contrast, develops through excessive expectations placed on children by parents and/or teachers (Ishida, 2012a). These negative experiences include receiving strong instructions (e.g., “Correct your stuttering. You cannot succeed in life if you continue to stutter.”) and inappropriate praise (e.g., “Your speech without stuttering is good. You should speak like this from now on.”). Thus, these repeated negative experiences act as extrinsic rewards that reinforce the excessive need for approval from others and increase anxiety, which may then result in increased stuttering (Freeman & Silver, 1989). Therefore, parents and teachers should not impose excessive expectations on children who stutter. Pleasure from past, present and future challenges and events may be effective in the development of PIL/ikigai and a well-balanced secretion of neurotransmitters related to the development of emotions (Ishida, 2012a). However, this does not suggest that an individual should perform new challenges and engage in new events continuously throughout their lives. That is, when the individual no longer receives pleasure and comfort from specific experiences, they should stop until interested in the experience again. This process may help individuals find and develop their PIL/ikigai sooner.

In the following paragraph, we provide a brief note and summary on previous findings on PIL/ikigai. Many people, including both stutterers and non-stutterers, understand the significance of the fact that variations of PIL/ikigai depend on intrinsically motivated individual interests, not on the interests of others. Some individuals, however, may desire excessive SD, which could lead to the notion that every person *should* or *must* achieve PIL/ikigai (Hosaka, 2012). These persons with excessive SD make an effort both to establish PIL/ikigai *faster* and to achieve *a higher level of PIL/ikigai than others* (Hosaka, 2012). These processes are based on the need for approval accompanied by anxiety and increased stuttering, which leads to an imbalanced secretion of emotion-related neurotransmitters. Therefore, the amount of time it takes for a person to find and develop their PIL/ikigai, as well as the level to which they have achieved it, should resist attempts to be made competitive and categorized.

The findings from the present review should be taken with a few limitations in mind. First, the proposal for reduced anxiety in stuttering is based on limited research and findings. In this paper, both positive experiences which may have influenced PIL/ikigai and negative experiences which may have influenced SD were based on studies focusing on experiences from infancy through adolescence. Recent studies have shown that the frontal lobe function develops over time (Craik & Grady, 2002). In addition, the association suggested in this study must be verified by a behavior evaluation of patients. Therefore, future studies should be performed based on the idea that the mind and body are integrated and that health may also be influenced by social attitudes.

## 3. Conclusion

The development of emotional states such as anxiety and comfort are related to the secretion of neurotransmitters and controlled by the prefrontal lobe. Thus, the most apparent problem for stutterers seems to be excessive anxiety caused by a perceived failure in conversing with others. Thus, the main cause of their stress and anxiety seems to perceived failure to articulately engage in conversations with others and gain SD. Purpose in life (PIL)/ikigai, a frontal lobe function which is more evolved in humans than other mammals, provides comfort and reduces both anxiety, even that during stressful situations, and the need for SD. Therefore, individuals including stutterers should engage in more positive than negative experiences in order to both develop PIL/ikigai and change neuronal networks through the repeated well-balanced secretion of neurotransmitters. This process could lead to a decrease in stuttering.
